# Detection of Prions in Blood of Cervids at the Asymptomatic Stage of Chronic Wasting Disease

**DOI:** 10.1038/s41598-017-17090-x

**Published:** 2017-12-08

**Authors:** Carlos Kramm, Sandra Pritzkow, Adam Lyon, Tracy Nichols, Rodrigo Morales, Claudio Soto

**Affiliations:** 1 Mitchell Center for Alzheimer’s disease and Related Brain Disorders, Dept. of Neurology, McGovern School of Medicine University of Texas Health Science Center at Houston, Houston, TX 77030 USA; 2 Universidad de los Andes, Facultad de Medicina, Av. San Carlos de Apoquindo, 2200 Las Condes, Santiago Chile; 3 Veterinary Services Cervid Health Program, APHIS, United States Department of Agriculture, Fort Collins, CO 80526 USA

## Abstract

Chronic wasting disease (CWD) is a rapidly spreading prion disorder affecting captive and free-ranging cervids. The zoonotic potential of CWD is unknown, as well as the mechanism for its highly efficient transmission. A top priority to minimize further spreading of this disease and its potential impact on environmental prion contamination is the development of a non-invasive, sensitive, and specific test for *ante-mortem* detection of infected animals. Here, we optimized the protein misfolding cyclic amplification (PMCA) assay for highly efficient detection of CWD prions in blood samples. Studies were done using a blind panel of 98 field-collected samples of whole blood from codon 96 glycine/glycine, captive white-tailed deer that were analyzed for prion infection *post-mortem* by immunohistochemistry (IHC). The results showed a sensitivity of 100% in animals with very poor body condition that were IHC-positive in both brain and lymph nodes, 96% in asymptomatic deer IHC-positive in brain and lymph nodes and 53% in animals at early stages of infection that were IHC-positive only in lymph nodes. The overall mean diagnostic sensitivity was 79.3% with 100% specificity. These findings show that PMCA might be useful as a blood test for routine, live animal diagnosis of CWD.

## Introduction

Prion diseases are fatal infectious diseases affecting humans and various species of mammals, including sheep, goats, mink, cervids, cattle and felines. Although prion diseases are rare in humans, the reported animal-to-human transmission and the increasing prevalence of some animal diseases present important problems for public and animal health. The most common animal prion disease is scrapie, a disorder of sheep and goats that has been recognized for centuries and has become an endemic problem nearly worldwide. Currently the most worrisome animal diseases are bovine spongiform encephalopathy and chronic wasting disease (CWD). CWD is the only known prion disease of wild animals; it is highly contagious and the exact origin, prevalence and mechanisms for transmission remain incompletely understood^1–3^. The disease has been rapidly expanding geographically and now affects 24 states in the USA, two Canadian provinces, South Korea and Norway. The risk of transmission of CWD to other animal species is unknown and surveillance to detect the infection in non-cervid species is limited. Much effort has been dedicated to analyze the potential for CWD transmission to humans^4^. To date, no evidence of CWD transmission to humans has been documented. Nevertheless, the US Centers for Disease Control and Prevention (CDC) strongly advise to keep cervid derived prions out of the human food chain. The potential transmission of CWD prions to humans has no clear answer yet, due to limitations with animal models and the lack of detailed knowledge concerning the factors in the host and the mechanisms controlling the species barrier.

CWD affects various species of captive and free-ranging cervids, including mule deer, white-tailed deer, elk, reindeer, sika, muntjac and moose^3^. The clinical symptoms include progressive weight loss, difficulties in movement, behavioral changes, decreased interactions with other animals, lethargy, lowering of the head, tremors, nervousness and repetitive walking in set patterns^5^. As with other prion diseases, brain abnormalities include spongiform degeneration, neuronal loss, glial activation and the accumulation of a protease-resistance version of the prion protein, termed PrP^Sc^. PrP^Sc^ is mostly found in the CNS, but minute levels have also been detected in various peripheral tissues^6^. Compelling evidence indicates that PrP^Sc^ is the sole component of the infectious agent. Therefore, the hallmark event in the disease is the conversion of the normal isoform of the prion protein (termed PrP^C^) into the misfolded form, which is templated by PrP^Sc^ present in the infectious material^7,8^.

The efficient propagation of CWD in captive and free-ranging cervids suggests that transmission may involve retention and enrichment of infectious prions in the environment^9–12^. Studies have reported instances of CWD recurrence upon reintroduction of animals to premises previously exposed to infected animals many years before^13^. Infectious prions can enter the environment through saliva, feces, urine, blood or placenta tissue from infected animals, as well as by decomposing carcasses from dead animals^14–16^. Importantly, CWD prion shedding in excreta has been shown to occur many months before onset of clinical disease and the cumulative amount of prions released from infected, but not yet sick, animals could be very large^11^. Various reports have shown that infectious prions bind tightly to soil and remain infectious after years in this material^17–19^, suggesting that environmental contamination may play an important role in CWD spreading. This conclusion is further supported by recent findings showing that prions bind to a variety of components of the environment, including plants, and remain infectious by oral administration^20^. Genetic analyses have shown that various polymorphisms in the gene encoding the prion protein (*Prnp*) affects CWD susceptibility in cervid populations. In white-tailed deer, a polymorphism at position 96, either a glycine (G) or a serine (S), influences CWD susceptibility, rate of disease progression and the extent of peripheral distribution of PrP^Sc^ (ref.^21^). The most common genetic makeup of wild white-tailed deer affected by CWD is GG at position 96, with the presence of at least one copy of the S-allele reducing the disease susceptibility^21^.

An important goal to minimize the further spreading of CWD is the development of an assay for highly sensitive, non-invasive, and inexpensive *ante-mortem* detection of prions in infected, but not clinically sick animals. Currently, CWD is definitively diagnosed by *post-mortem* examinations of brain, brain stem and lymphoid tissues^3^. For this purpose, brain, brain stem at the obex, and medial retropharyngeal lymph nodes (MRPLN) samples are collected *post-mortem*, and analyzed by immunohistochemistry (IHC), ELISA, and/or western blot (WB) to detect PrP^Sc^ (ref.^22^). It is estimated that *post-mortem* IHC analysis (considered the gold standard for CWD regulatory testing in the USA) can identify the presence of infectious prions in the MRPLN and obex, as early as three and six to nine months after infection, respectively^23^. Recently, IHC detection of PrP^Sc^ in rectal biopsy was evaluated as an *ante-mortem* test^24–26^. The diagnostic sensitivity of this assay was variable depending on the genotype of the animal and the disease progression at the moment of sample collection, ranging from 36% to 100%^25^. Particularly, low sensitivity was observed in animals at the early stage of infection when the obex was negative for PrP^Sc^, and positive staining was only detected in MRPLN^25^. Furthermore, although the rectal biopsy is relatively simple, it is an invasive, and expensive procedure.

Like scrapie, CWD has a wide distribution of prion infection in peripheral tissues and biological fluids. Indeed, infectivity bioassays in deer or transgenic mice expressing cervid prion protein (PrP) have shown the presence of infectious materials in a large variety of tissues, including CNS tissues, peripheral nerves, lympho-reticular organs, gastro-intestinal tissues and skeletal muscle^2^. Infectivity was also found in various biological and excretory fluids, including blood, saliva, urine and feces^2,3^. These findings offer hope for the development of an assay that can detect prions in easily accessible biological fluids. However, it is likely that the quantity of PrP^Sc^ present in these fluids is very small, orders of magnitude under the level of sensitivity of the commonly used WB and ELISA assays. In recent years, the implementation of techniques for extremely high sensitive detection of prions in biological fluids has been a major breakthrough in diagnosing various prion diseases^27–30^. These techniques rely on the amplification of PrP^Sc^ and include the protein misfolding cyclic amplification (PMCA) and a variant of it termed real-time quaking induced conversion (RT-QuIC)^31,32^. Both of these procedures take advantage of the PrP^Sc^ capacity to seed the conversion of PrP^C^ into the abnormal form and employ a mechanical force to fragment the PrP^Sc^ aggregates leading to the cyclic amplification of the prion replication process. These procedures enable specific detection of very small quantities of PrP^Sc^ in tissues and biological fluids, likely approaching the levels of single particles of PrP^Sc^. Both PMCA and RT-QuIC have been used to detect with high sensitivity and specificity CWD prions in various tissues, fluids and excreta^14,15,20,33–37^. Arguably, blood offers the best opportunity for non-invasive and routine testing of large populations of cervids for potential prion infection. PMCA has been extensively used in the past to detect various human and animal prions in blood samples collected at both the symptomatic and asymptomatic stages of the disease^38–44^. However, until now, it has not been used to detect CWD prions in blood samples collected from naturally infected deer. The main goal of this study was to optimize PMCA for sensitive detection of CWD prions in blood and study the utility of the technique in field collected samples for *ante-mortem* diagnosis of CWD-infected animals.

## Results

**Figure 1:**
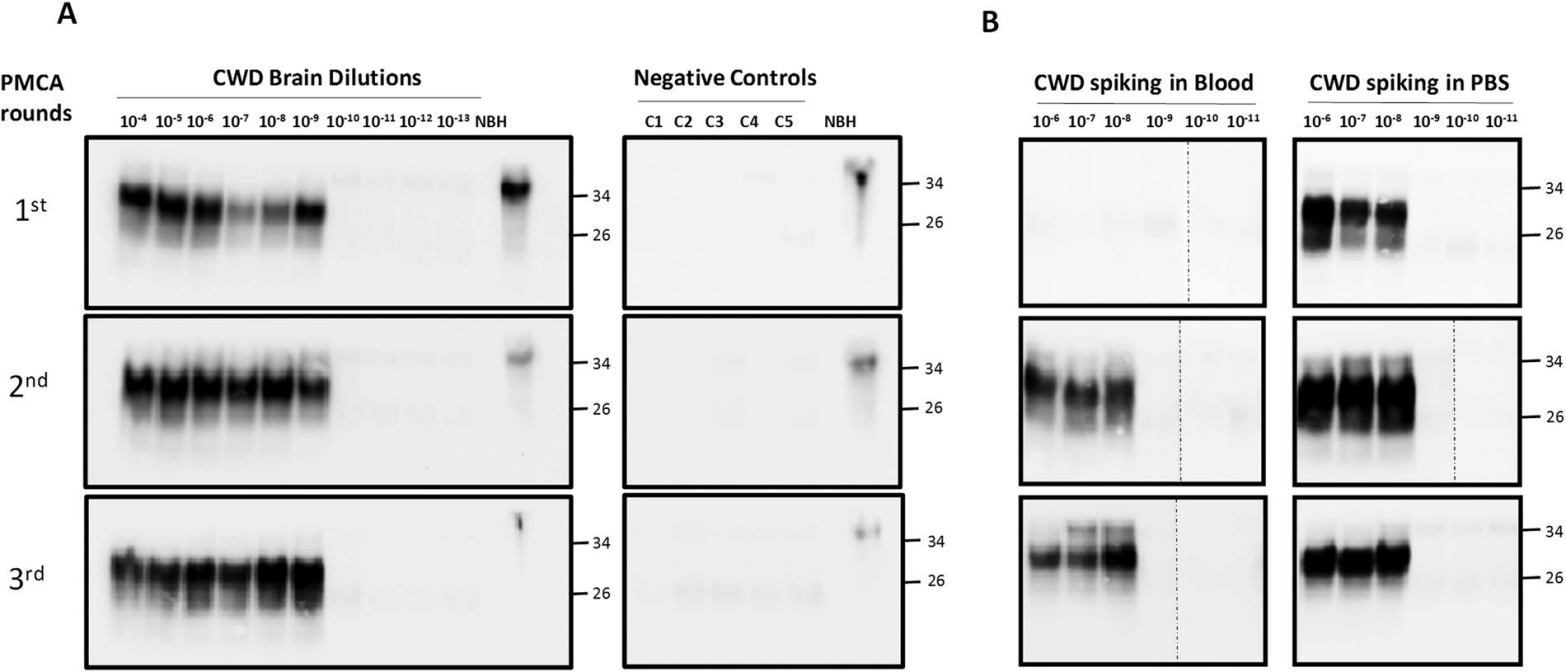
PMCA of CWD prions. (**A**) To optimize CWD PrP^Sc^ amplification by PMCA and determine the limit of detection, brain extracts from CWD sick animals was serially diluted (10^−4^ to 10^−13^) in buffer and subjected to various consecutive rounds of 144 PMCA cycles. As negative controls, 5 tubes (C1 to C5) in which PMCA was done without CWD brain homogenate were used to control for possible cross-contamination. After each PMCA round, an aliquot of 10 µL was taken to analyze for PrP^Sc^ signal by western blot using the 6H4 anti-PrP antibody. All samples, except the normal brain homogenate (NBH), were treated with 10 µg/mL of PK for 1 h at 37 °C, before western blotting to differentiate PrP^Sc^ from PrP^C^. (**B**) Whole blood from a healthy deer was spiked with CWD brain homogenate at distinct final dilutions (10^−6^ to 10^−11^). The same dilutions were spiked in buffer (PBS) as control (right panel). After processing by high-speed centrifugation in the presence of sarkosyl (as described in Methods), samples were subjected to three consecutive rounds of PMCA. The PrP^Sc^ signal was assessed by Western blot analysis after PK digestion. NBH refers to the transgenic normal (healthy) brain homogenate, used as migration control marker. Dashed lines in some of the blots indicate splicing, done to remove unrelated lanes. Numbers in the right indicate the position of molecular weight markers.

To optimize PMCA for ultra-sensitive detection of CWD PrP^Sc^ and analyze the limit of detection of the assay, we first serially diluted a 10% brain homogenate from a deer with confirmed CWD and performed serial rounds of PMCA. Under our optimized conditions, maximum amplification was obtained after only 1 round of 144 PMCA cycles, with a limit of detection equivalent to a 10^−9^ dilution of the brain extract (Fig. 1A). The exact limit of detection depends on the amount of PrP^Sc^ present in the particular animal and on the brain region utilized, which usually ranges between 10^−8^ and 10^−11^. This sensitivity is similar to that reported previously for rodent and human prion samples^43,45–47^ and is on the range of detection of single PrP^Sc^ particles^45,46^. None of the unseeded PMCA assays gave any protease-resistant PrP^Sc^ signal (Fig. 1A). Next, we spiked the same dilutions of CWD brain homogenate into healthy, CWD-free deer blood, processed the samples through sarkosyl precipitation (as indicated in the Methods section) and performed the PMCA assay. Maximum amplification was achieved in the second round of PMCA (Fig. 1B), indicating that despite the sample processing the presence of blood components still interfered with the assay. Nevertheless, the limit of detection was the same as the samples spiked in buffer (Fig. 1B).

**Figure 2:**
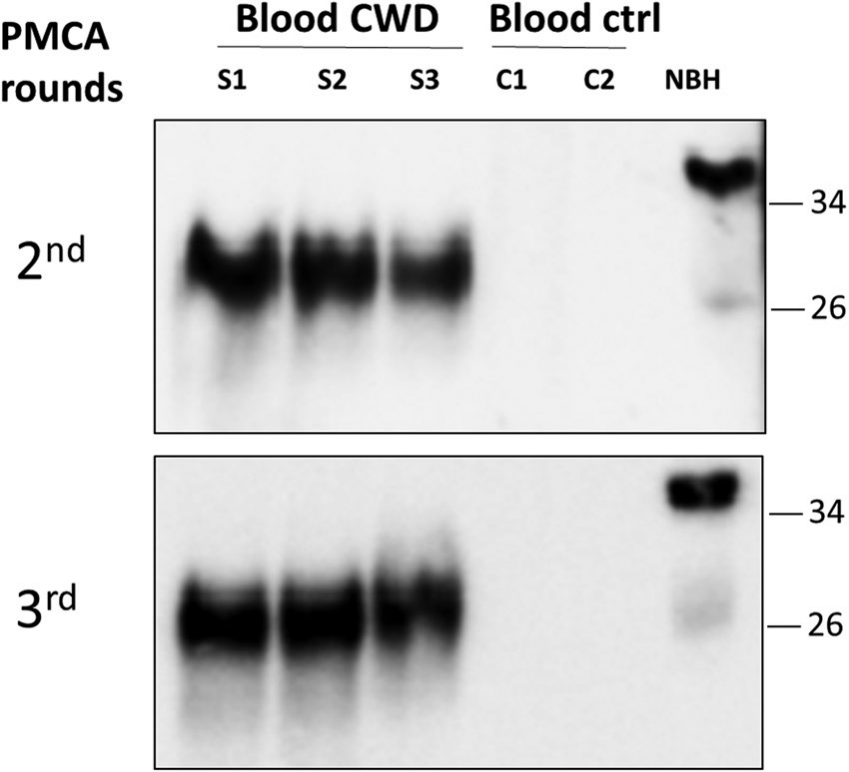
PrP^Sc^ detection in blood of animals showing clinical symptoms of CWD. Representative samples of whole blood (80 µL) from three white-tailed deer positive for PrP^Sc^ in obex and MRPLN as examined by IHC and presenting with poor body condition (S1, S2, S3), were analyzed by PMCA. As controls, blood from two deer from a CWD-free control herd (C1 and C2) were analyzed in parallel. Samples were subjected to three serial rounds of PMCA and the results of rounds 2 and 3 are shown. The PRC1 anti-PrP antibody was used for western blotting immunodetection. All samples were treated with PK, except the transgenic mice normal brain homogenate (NBH), used as migration control. Numbers in the right indicate the position of molecular weight markers.

**Figure 3:**
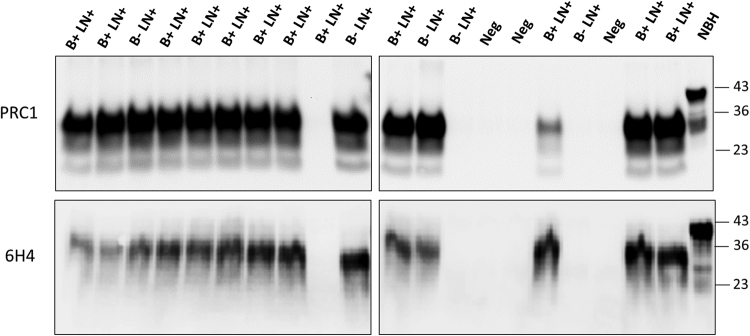
Blind study of PrP^Sc^ detection in blood of CWD-infected, but asymptomatic animals. Representative samples of whole blood (200 µL) from asymptomatic white-tailed deer, were collected from either CWD-free or CWD-infected herds. Samples analyzed included 49 animals that were positive for PrP^Sc^-staining in both the MRPLN and the obex via IHC (B + LN + ), 34 animals that were positive for PrP^Sc^-staining only in the MRPLN via IHC (B-LN + ) and 10 that were negative in both brain and MRLPN. The figure shows representative samples from 12 B + LN + , 5 B-LN + and 3 B-LN- (Neg). The entire set of samples was analyzed independently by two different investigators in duplicate. Top and bottom panels show the results from the two different investigators which developed their western blots using two distinct anti-PrP antibodies (PRC1 and 6H4). Samples were subjected to three serial rounds of PMCA and the results obtained in the third round are shown. All samples were treated with PK, except the transgenic mice normal brain homogenate (NBH), used as migration control. Numbers in the right indicate the position of molecular weight markers.

We analyzed blood samples from five deer showing very poor body condition, which were positive in both obex and MRPLN by IHC, along with three replicates coming from a pool of blood from seven animals with negative IHC. The results showed a clear detection of PrP^Sc^ signal in all of the samples from CWD-infected animals and none in the controls (Fig. 2 shows representative samples from this set), indicating a 100% sensitivity and specificity in this small group of samples. Next, we analyzed a panel of 93 whole blood samples provided blindly by the United States Department of Agriculture (USDA) scientists from captive white-tailed deer. For the studies included in this article we focused exclusively on deer harboring the most common polymorphic variant (GG at position 96). These samples were from animals which did not show any gross clinical or behavioral abnormalities and included 10 samples collected from farms free of CWD, 49 that were IHC positive in both obex and MRPLN and 34 that were not detected in obex and IHC-positive only in MRPLN. Samples were processed independently by two different investigators who ran each sample in duplicate. Figure 3 shows the results for representative samples from each group, which were detected using two different anti-PrP antibodies. For the samples from animals that were PrP^Sc^ positive in obex and MRPLN we detected most of them (47 out of 49) as positives after two or three rounds of PMCA, whereas none of the 10 samples coming from CWD-free deer gave any signal (Fig. 3). Conversely, 18 of the 34 deer that were only IHC positive in the MRPLN gave a positive signal in the PMCA assay. Overall, the assay reached a sensitivity of 100% in symptomatic animals, 96% in deer positive in obex and MRPLN and 53% in animals that were only positive in MRPLN (Table 1). The overall mean diagnostic sensitivity was 79.3% and specificity was 100%.

**Table 1:** Summary of the results obtained in a blind study of detection of CWD prions in blood samples from white-tailed deer at various stages of CWD.

CWD status	Total number of animals	PMCA positive^1^	% Correct diagnosis
Symptomatic (B + LN + )	5	5	100%
Asymptomatic (B + LN + )	49	47	97%
Asymptomatic (B- LN + )	34	18	53%
Negative (B- LN-)	10	0	100%

^1^Samples were declared as positive in PMCA if at least one of the replicates gave a protease-resistant PrP^Sc^ signal in any of the PMCA rounds analyzed.

**Figure 4:**
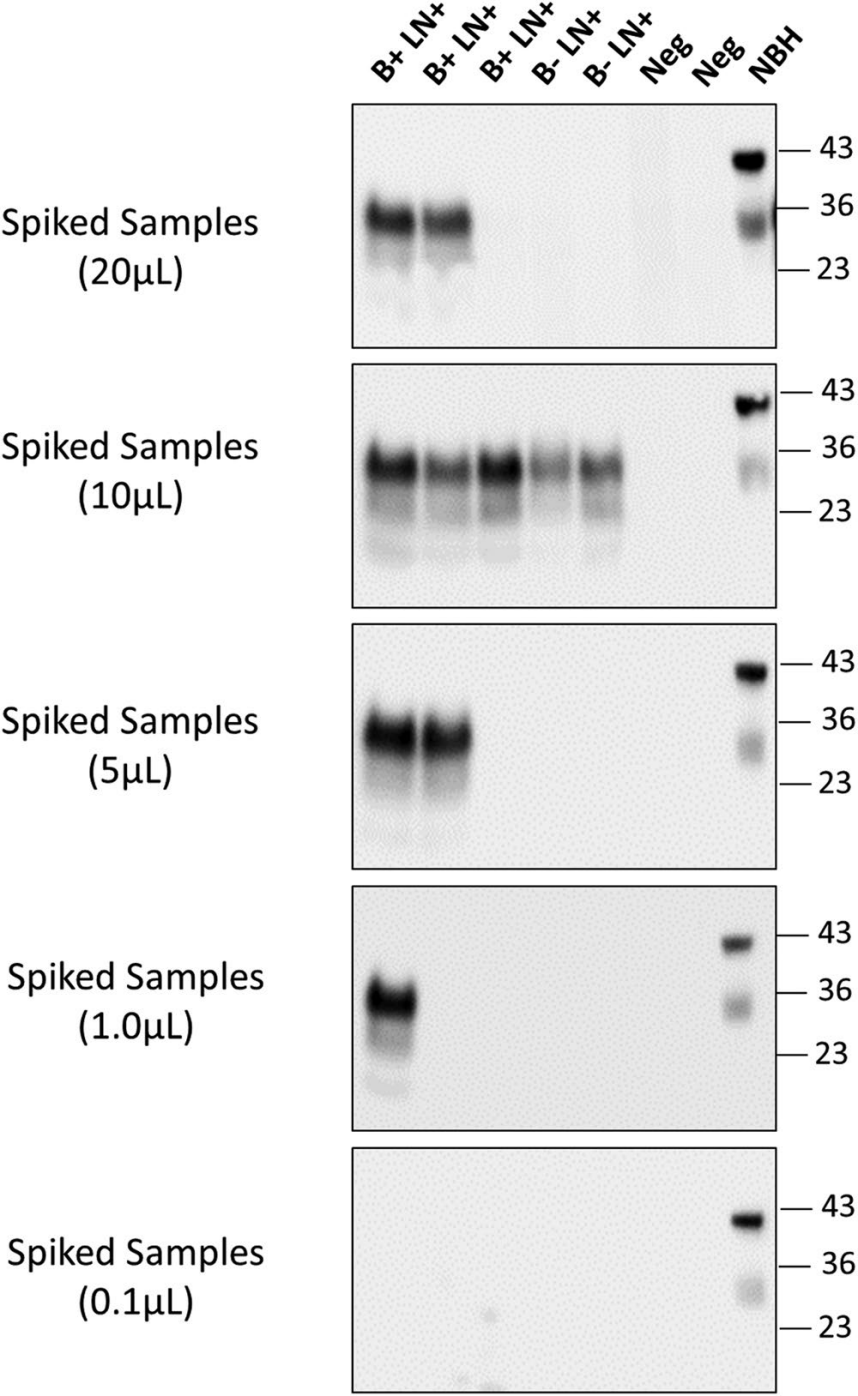
Detection of PrP^Sc^ in small volume of blood and removal of pre-cleaning step. To estimate the minimum amount of blood needed for detection, different volumes (20, 10, 5, 1 and 0.1 µL) of whole blood from five representative samples that were positive in the blinded study (3 B + LN + and 2 B-LN + ), were directly added to a 10% brain homogenate from cervid transgenic mice. As controls we used two samples from CWD negative animals. Samples were subjected to three sequential rounds of PMCA and PrP^Sc^ detected by Western blot using the PRC1 antibody. The figure shows the results of the third round of PMCA. As before, all samples were treated with PK, except the transgenic mice normal brain homogenate (NBH), used as migration control. Numbers in the right indicate the position of molecular weight markers.

As we previously described for the detection of human PrP^Sc^ in blood from vCJD patients, it is possible to eliminate the time-consuming and effort-demanding pre-cleaning step to remove inhibitors of the PMCA reaction by using small volumes of blood samples^43^. In order to evaluate whether this strategy may also work with CWD blood, we took five random samples from those previously shown to be positive with the standard assay, along with two negatives, and added different small volumes of whole blood directly into the PMCA reaction. As shown in Fig. 4, using 10 µl of blood showed perfect sensitivity and specificity in this set of samples analyzed, whereas larger or smaller volumes only enabled correct detection in some of the samples. These results may permit the implementation of a simpler procedure for PrP^Sc^ detection in CWD blood, leading to substantial savings of time and samples, but the robustness of the simplified assay needs to be confirmed by blind analysis of a larger number of samples.

## Discussion

A safe, non-invasive, sensitive and specific live animal test to identify cervids infected with CWD prions is a top priority to control further spreading of this emerging infectious disease. Availability of such a test could have many important applications, including: (i) screening of deer farms to remove infected animals before they can spread substantial amounts of prions into the environment; (ii) monitoring the efficacy of prion decontamination procedures; (iii) monitoring the extent of environmental prion contamination; and (iv) whole herd screenings for determination of CWD status.

Currently, the only definitive way to diagnose CWD is by *post-mortem* examination of brain and lymphoid tissues for the presence of PrP^Sc^ by IHC, the current gold standard for prion detection. Various attempts have been made to develop a test for *ante-mortem* detection of prions during the long, silent period between prion infection and the onset of clinical signs of the disease, which can encompass several months or years. One strategy that has been evaluated is palatine tonsil biopsy followed by IHC analysis^48^. However, this method is invasive and time consuming, and requires anesthesia that may lead to complications. An alternative possibility for *ante-mortem* CWD detection, is rectal biopsy followed by IHC analysis for protease resistant PrP^Sc^. Various independent studies have shown the efficacy of this procedure for pre-clinical detection of prions in CWD-infected animals^24–26^. In a collaborative study between the Canadian Food Inspection Agency and the USDA, a detailed analysis was done for the sensitivity and specificity of IHC detection of PrP^Sc^ in rectal biopsy samples^25^. For this study, samples were collected *post-mortem* from white-tailed deer in four CWD-infected herds. PrP^Sc^ detection was compared to the CWD infection rate estimated by IHC analysis of the obex and MRPLN. The overall diagnostic sensitivity of this assay was estimated at 68%, including all stages of the disease and all polymorphic variants analyzed^25^. The study also showed a clear correlation between disease progression, genotype at codon 96 and the presence of PrP^Sc^ signal in rectal biopsy samples. Disease progression was estimated from the presence and degree of PrP^Sc^ signal in obex as well as prion detection in MRPLN. In early disease progression (low obex grades), the diagnostic sensitivity of the rectal biopsy assay was 36% in samples from animals that did not have PrP^Sc^ in brain, but were positive in MRPLN^25^. The genotype at codon 96 also strongly influenced the outcome of the rectal biopsy IHC, with GG animals having a higher detection rate than GS and SS animals^25^. Sensitivity was slightly increased by using RT-QuIC instead of IHC, reaching an overall diagnostic sensitivity (including all disease stages and polymorphisms) of 70% in samples obtained by rectal biopsy^36^. In this study, sensitivity for RT-QuIC in rectal samples from animals negative in the obex was 25%, compared to only 8% using IHC. However, several false positives were found using this technique, decreasing specificity to 94%^36^. The same authors also analyzed the efficacy of RT-QuIC using nasal brushing as a less invasive sample collection compared to rectal biopsy. The results showed a diagnostic sensitivity of only 34%^49^, suggesting that this strategy is significantly less effective for cervid samples than human sCJD samples^50^.

In our blind study using field collected blood samples from white-tailed deer, we found a mean overall diagnostic sensitivity of 79.3%, with 100% specificity. Sensitivity for blood samples from CWD-infected animals at the pre-clinical stage of the disease was 96%, when were IHC positive in both the obex and the MRPLN. For similar samples in which only the MRPLN was IHC positive, sensitivity was reduced to 53%. Provided that the infection status of these animals was correctly determined by IHC and because of the extremely high sensitivity of PMCA, which is able to detect few particles of PrP^Sc^ aggregates^45,46^, these results suggest that at some stages of the pre-clinical disease, infected animals may not have PrP^Sc^ in blood. Since obtaining blood samples is relatively non-invasive, a much more accurate *ante-mortem* laboratory diagnosis of CWD could be done by repeating the PMCA test multiple times in live animals. This scheme is useful because sensitivity approaches 100% as the disease progress. Nevertheless, further studies need to be done employing larger number of samples in which the status of prion infection has been confirmed by IHC and a biochemical procedure (western blot or ELISA), as well as analyzing the efficacy of the test in animals harboring the less frequent *Prnp* genotypes (such as GS and SS at position 96). Studies should also be done in blood samples longitudinally collected from experimentally infected deer to determine the earliest time in which PrP^Sc^ can be detected by PMCA and the dynamic changes on the PrP^Sc^ levels present in blood during the incubation period. Our results suggest that PMCA may provide a suitable platform for rapid prion diagnosis in farmed and wild cervids. Early and non-invasive CWD prion detection could not only help to control severely affected premises, but also be useful for surveillance of areas where CWD cases are not yet known to occur.

## Materials and Methods

### Study Populations

The samples utilized in the present study were collected by scientists from the USDA from captive, codon 96 GG (glycine, glycine), white-tailed deer (*Odocoileus virginianus*) from a CWD-free control herd or depopulated CWD-infected herds within the United States. Blood samples were collected venously in EDTA tubes *ante-mortem*, either from animals within a chute, or when chemically immobilized for euthanasia. Samples from a total of 98 deer were utilized, ten of which were from a CWD-free research herd. 34 animals were positive for PrP^Sc^-staining only in the MRPLN via IHC, while asymptomatic and appearing to be in good health. 54 animals were positive for PrP^Sc^-staining in both the MRPLN and the obex via IHC. These animals were also asymptomatic and in good body condition, with the exception of five individuals that were potentially at the beginning of the symptomatic disease phase (very poor body condition, poor coat and extreme emaciation).

### Sample Collection

After euthanasia, animals from the CWD-infected herds were aged either by herd records or by estimation based on visual inspection of the teeth by a wildlife biologist. Brainstem, at the level of the obex and MRPLN, were collected post-mortem utilizing standard USDA regulatory sample collection methods (For information, see https://www.aphis.usda.gov/animal_health/animal_diseases/cwd/downloads/cwd_program_standards_2014.pdf). Blood was collected from the jugular vein in commercial EDTA blood tubes and stored at −80 °C until used. Regulatory samples of obex and MRPLN were collected and stained *post-mortem* from the euthanized animals for protease-resistant PrP by IHC, as previously described^51^.

### Processing of Blood Samples

Frozen samples of whole blood were processed to reduce proteins and other components that interfered with the PMCA reaction, as previously described^28^. Briefly, 200 µL of sample were mixed and incubated with 1 volume of 20% sarkosyl for 1 h at room temperature. Thereafter, samples were centrifuged at 100,000 × g for 1 h at 4 °C, supernatant was discarded and pellet washed in 400 µL of PBS. Tubes were centrifuged again at 100,000 × g for 30 min at 4 °C. The pellet was resuspended directly in 10% brain homogenate from transgenic mice expressing cervid PrP^C^, as described below. Each sample was simultaneously processed in four independent replicates by two different investigators. For some experiments, 10 µL of whole blood were directly added to the PMCA reaction without sarkosyl processing.

### PMCA Assay

The PMCA reaction was carried out as previously described^28,47^, using brain homogenate from mice expressing cervid PrP^C^ (Tg1536) as substrate. These animals were kindly provided by Dr. Glenn Telling (Colorado State University) and a colony was established in our facility. Brain substrate was prepared at a concentration of 10% (weight/volume) in conversion buffer (PBS supplemented with 150 mM NaCl and 1% Triton X-100, 0.025% Digitonin (Invitrogen #BN20061) and 3mM EDTA (Promega Cat V4231)) with protease inhibitors (Complete, Roche). Debris were removed by a low speed centrifugation (800 × g, 1 min, 4 °C) and brain homogenates were stored frozen at −80 °C until further use.

For PMCA, samples were subjected to 144 cycles of PMCA in 0.2 mL tubes (Eppendorf, cat. N. 951010022) containing 3 teflon beads (Hoover precision products) by either resuspending sarkosyl extraction pellets in PMCA substrate or direct spiking of brain homogenates or blood samples. Each cycle consisted of a 29 min and 40 s incubation at 37 °C followed by a 20 s pulse of sonication set at a potency of 110–120 W, using a Misonix microsonicator (Model S4000) equipped with a titanium horn. Subsequent rounds of 96 PMCA cycles were done by taking an aliquot of the amplified material, which was diluted 10-fold into fresh transgenic mice brain homogenate. After each round of PMCA, samples were taken for detection of PrP^Sc^ with the 6H4 or the PRC1 anti-PrP antibodies via Western blot after digestion with proteinase K, as described^28,47^. All precautions were taken to avoid cross-contamination as illustrated in previous publications^28^.

### Ethics Statement

This project does not involve animal experimentations. Blood samples from white-tailed deer were collected previously for other purposes by Dr Tracy Nichols. We have USDA approval to receive and work with these samples. As substrate for PMCA, we used brains from transgenic mice expressing deer PrP^C^. The procedures for breeding, manipulations and euthanasia of these transgenic mice was performed following NIH guidelines and approved by the Animal Welfare Committee of the University of Texas Medical School at Houston (protocol AWC-16-0057).
